# Case report: high-degree atrioventricular block associated with takotsubo cardiomyopathy: What is the right timing for pacemaker implantation?

**DOI:** 10.1093/ehjcr/ytag293

**Published:** 2026-05-07

**Authors:** Sacha Jourdin, Gabriel Chevrot, Tej Chalbia, Nicolas Mansencal, Marie Hauguel-Moreau

**Affiliations:** Department of Cardiology, Ambroise Paré Hospital, Assistance Publique-Hôpitaux de Paris (AP-HP), Centre de Référence des Cardiomyopathies et des Troubles du Rythme Cardiaque Héréditaires ou Rares, Université de Versailles-Saint Quentin (UVSQ), 9 avenue Charles de Gaulle, 92100 Boulogne-Billancourt, France; Department of Cardiology, Ambroise Paré Hospital, Assistance Publique-Hôpitaux de Paris (AP-HP), Centre de Référence des Cardiomyopathies et des Troubles du Rythme Cardiaque Héréditaires ou Rares, Université de Versailles-Saint Quentin (UVSQ), 9 avenue Charles de Gaulle, 92100 Boulogne-Billancourt, France; Department of Cardiology, Ambroise Paré Hospital, Assistance Publique-Hôpitaux de Paris (AP-HP), Centre de Référence des Cardiomyopathies et des Troubles du Rythme Cardiaque Héréditaires ou Rares, Université de Versailles-Saint Quentin (UVSQ), 9 avenue Charles de Gaulle, 92100 Boulogne-Billancourt, France; Department of Cardiology, Ambroise Paré Hospital, Assistance Publique-Hôpitaux de Paris (AP-HP), Centre de Référence des Cardiomyopathies et des Troubles du Rythme Cardiaque Héréditaires ou Rares, Université de Versailles-Saint Quentin (UVSQ), 9 avenue Charles de Gaulle, 92100 Boulogne-Billancourt, France; Department of Cardiology, Ambroise Paré Hospital, Assistance Publique-Hôpitaux de Paris (AP-HP), Centre de Référence des Cardiomyopathies et des Troubles du Rythme Cardiaque Héréditaires ou Rares, Université de Versailles-Saint Quentin (UVSQ), 9 avenue Charles de Gaulle, 92100 Boulogne-Billancourt, France

**Keywords:** Takotsubo Cardiomyopathy, Atrioventricular block, Pacemaker, Case report

## Abstract

**Background:**

Takotsubo cardiomyopathy (TCM) is a transient left ventricular systolic dysfunction typically triggered by emotional or physical stress. High-degree atrioventricular (AV) block is a rare but significant complication of TCM. Its occurrence raises complex management questions, particularly regarding the appropriate timing for permanent pacemaker implantation given the possible reversible nature of both conditions.

**Case Summary:**

We report the case of a 65-year-old woman with diabetes who was admitted for syncope and found to have a complete AV block. Echocardiography and cardiac MRI showed apical akinesia and basal hyperkinesia, consistent with TCM. Coronary angiography revealed no obstructive coronary artery disease. Despite isoprenaline infusion and temporary pacing, the AV block persisted. A dual-chamber permanent pacemaker was implanted on day 6. By day 15, left ventricular ejection fraction had normalized, and at one-month follow-up, sinus rhythm had recovered, though a left bundle branch block persisted.

**Discussion:**

A comprehensive review of Pubmed database reported 22 cases of TCM associated with high-degree AV block. Among them, 31.8% of patients experienced AV conduction recovery, mostly younger individuals. However, most of them had persistent AV block beyond one month, justifying pacemaker implantation. Our findings suggest that while permanent pacing is often necessary, a short observation period may be appropriate in haemodynamically stable, younger patients. Given the absence of formal guidelines, individualized management is essential, and further research is needed to optimize therapeutic strategies for this uncommon association.

Learning points

- Takotsubo cardiomyopathy may be associated with high-degree atrioventricular block, though causality remains unclear.
- In Takotsubo cardiomyopathy with high-degree atrioventricular block, sinus rhythm may recover, requiring optimal timing of pacemaker implantation.

## Introduction

Takotsubo cardiomyopathy (TCM) is a transient systolic dysfunction of the left ventricle. Its pathophysiology remains unknown. Hypotheses are proposed, including coronary artery spasm, microcirculatory dysfunction, and a massive catecholamine surge.^1^ Severe complications can occur: heart failure, cardiogenic shock, systolic anterior motion and mitral regurgitation, thromboembolic events related to intraventricular thrombus, and arrhythmias.^2^ High degree atrioventricular (AV) block associated with TCM is rare. When occurring, AV block associated with TCM management emphasizes the question of pacemaker implantation timing, given the transient nature of TCM.

We present a case report of a female patient with a high degree of AV block associated with TCM treated with pacemaker implantation and discuss the right timing for pacemaker implantation.

## Summary figure

**Figure:**
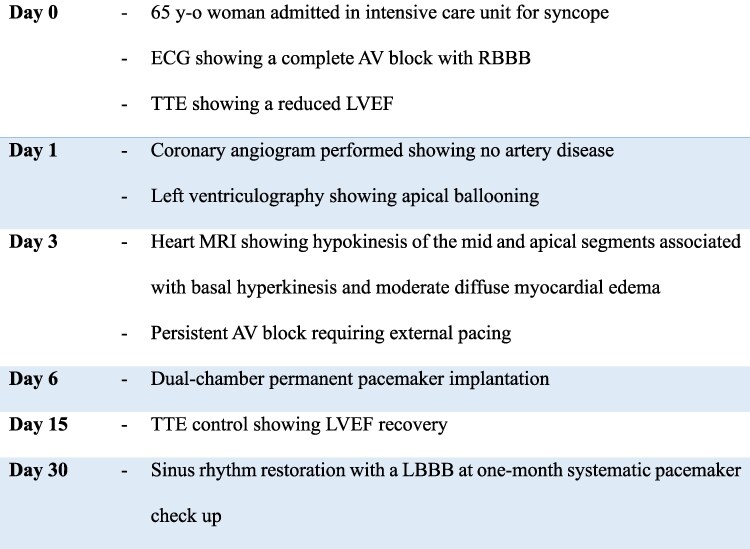


## Case presentation

We report the case of a 65-year-old female with a history of diabetes, admitted to the emergency department for syncope. She complained of asthenia for 1 week.

**Figure 1:**
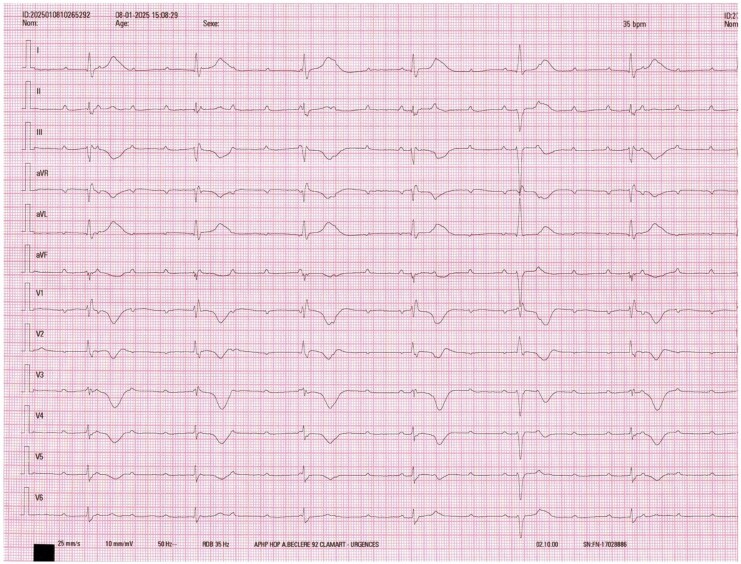
Electrocardiogram at admission showing a high degree atrioventricular block.

She did not report any chest pain or dyspnoea, nor any physical or emotional stressor. On admission, her blood pressure was 110/74 mmHg, pulse rate 27 bpm, oxygen saturation 98%, and temperature 36.8°C. Physical examination was normal. The electrocardiogram (ECG) revealed a complete AV block with a heart rate of 35 rate per minute (RPM) with large QRS complexes indicating escape rhythm (*Figure 1*). On presentation, serum lactate was normal (1.7 mmol/l), troponin US and NTproBNP were high (597 ng/l (*N* < 14 ng/l) and 2998 ng/l (<125 ng/l), respectively), without elevation of creatine kinase nor serum potassium abnormality, with a normal kidney function and a negative Lyme borreliosis serology.

**Figure 2:**
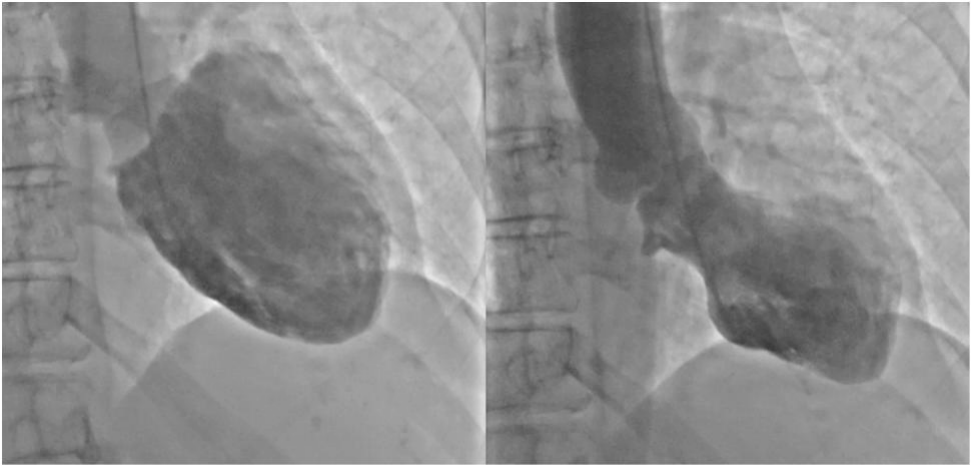
Left ventriculogram showing an apical ballooning consistent with Takotsubo cardiomyopathy.

Transthoracic echocardiography (TTE) showed a left ventricular ejection fraction (LVEF) of 15%, with akinesia of the apical segments and compensatory hyperkinesis of the basal segments, resembling TCM. Coronary angiogram revealed no significant coronary artery lesions, with ventriculography findings suggestive of TCM (*Figure 2*). Cardiac magnetic resonance imaging (MRI) performed at day 3 showed hypokinesis of the mid and apical segments associated with basal hyperkinesis and moderate diffuse myocardial oedema, consistent with TCM in recovery.

**Figure 3:**
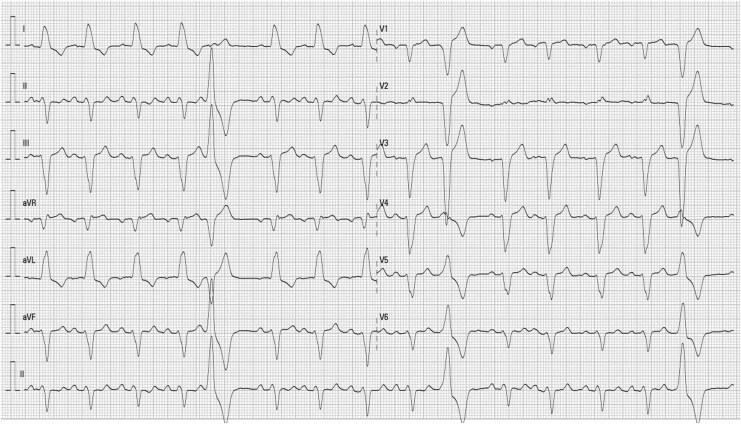
One-month electrocardiogram showing a sinus rhythm with a persistent left bundle branch block.

Isoprenaline was initiated (2 µg/min) on day 1 because of symptomatic bradycardia, after having ruled out left ventricular outflow tract obstruction with TTE. Despite isoprenaline (up to 10 µg/min), a persistent high degree AV block with profound bradycardia (nadir 17 bpm) required urgent implantation of a temporary transvenous pacing lead. Given the persistence of high-degree AV block on day 6 with a total pacemaker dependency, a dual-chamber permanent pacemaker was implanted, and the patient was discharged. TTE performed on day 15 showed improvement of the LVEF up to 55%, confirming the TCM. At 1-month follow-up, pacemaker interrogation showed regression of permanent AV block, with a regular sinus rhythm, a persistent left bundle branch block (LBBB) (*Figure 3*), and a rate of ventricular pacing of 5% (possibly paroxysmal complete AV block).

## Discussion

The association between TCM and complete AV block is rare, reported in 2.9% of cases of TCM.^3^ The temporal relationship between these conditions remains unclear, raising questions about causality when they occur simultaneously. While several cases report high degree AV block as a trigger of TCM, the simultaneous presentation of both entities complicates causal interpretation and suggests that AV block may also be a complication. In the present case, the clinical course provides arguments against this hypothesis. Notably, LVEF recovered earlier (day 15) than AV conduction, which only partially recovered at one month. Cardiac MRI demonstrated diffuse myocardial oedema, which may have extended to the conduction tissue and contributed to prolonged AV block. TCM predominantly affects elderly women, a population in which degenerative conduction system disease is more prevalent. Thus, underlying vulnerable AV conduction system may be unmasked or exacerbated by the myocardial disturbances associated with TCM, explaining the persistence of AV block despite recovery of LVEF. While AV block may trigger TCM in selected cases, our case may support AV block as a complication of TCM. This uneasy distinction has important clinical implications, particularly regarding the timing of permanent pacemaker implantation, as recovery of AV conduction may occur late, even after normalization of LVEF.

Several mechanisms are proposed to explain conduction disturbances in TCM. The catecholamine surge observed in TCM could induce vasospasm of small coronary artery branches. This could lead to transient ischaemia and conduction block. Another proposed mechanism involves reduced coronary perfusion due to left ventricular dyskinesis, impairing blood supply to the conduction system and resulting in AV block. A third hypothesis suggests that prolonged ischaemia due to myocardial stress could lead to fibrosis of the conduction pathways.^4^

A comprehensive literature search of the PubMed database (up to July, 25, 2025, including ‘Tako Tsubo cardiomyopathy’, ‘Takotsubo syndrome’, ‘stress cardiomyopathy’, ‘atrioventricular block’, ‘heart block’, ‘conduction disorder’, and ‘arrhythmia’) identified 22 cases of TCM associated with high-degree AV block (*Table 1*). Their main characteristics are summarized in *Table 2*. Seven (31.8%) patients recovered sinus rhythm, 3 of them (42.9%) showing early recovery within 7 days, while 4 between 1 and 3 months after discharge. Most patients did not recover sinus conduction and underwent pacemaker implantation, often occurring within the first two weeks. Patients who recovered were younger (61 [61–64] vs. 80 [61–83] years old), suggesting a potential association between young age and reversibility of conduction disorders.

**Table 1:** Cases review of high degree atrioventricular block in Takotsubo cardiomyopathy

Name of the study	Gender	Age, y	Chest pain at initial presentation	Syncope at initial presentation	QRS enlargement	If QRS enlargement, LBBB	If QRS enlargement, RBBB	Troponin I or T elevation	Initial LVEF alteration	LVEF restoration	AV conduction recovery	Delay of AV conduction restoration, d	Pacemaker implantation	Delay of pacemaker implantation, d
Revilla-Marti *et al.*, 2021^5^	F	61	No	Yes	Yes	No	Yes	Yes	Yes	Yes	Yes	1	No	
Chadha *et al.*, 2013^6^	F	61	Yes	No	No			No	Yes	Yes	Yes	30	Yes	6
Sugiura *et al.*, 2013^7^	F	63	No	Yes	No			No	Yes	Yes	Yes	1	No	
Prasad *et al.*, 2021^8^	F	71	No	Yes	Yes	No	Yes	Yes	Yes	Yes	Yes	42	Yes	1
Kown *et al.* 2022^9^	F	60	Yes	Yes	Yes	No	Yes	Yes	Yes	Yes	Yes	60	Yes	11
Vavilis *et al.*, 2022^10^	F	57	Yes	No	Yes	Yes	No	Yes	Yes	Yes	Yes	1274	Yes	7
Vavilis *et al.*, 2022^10^	F	85	No	Yes	Yes	Yes	No	Yes	Yes	No	No		Yes	2
Afzal *et al.*, 2025^11^	F	62	No	Yes	Yes	No	Yes	Yes	Yes	Yes	No		Yes	3
Benouda *et al.*, 2012^12^	F	81	Yes	No	No			Yes	Yes	Yes	No		Yes	60
Benouda *et al.*, 2012^12^	F	69	Yes	No	No				Yes	Yes	No		Yes	270
Dubey *et al.*, 2023^13^	F	53	Yes	No	No			Yes	Yes	Yes	No		Yes	5
Dubey *et al.*, 2023^13^	F	73	No	Yes	No			Yes	Yes	Yes	No		Yes	5
El Battrawy *et al.*, 2019^14^	F	86	No	Yes					Yes	Yes	No		Yes	2
Inayat *et al.*, 2017^15^	F	59	No	Yes	Yes	No	Yes	Yes	Yes	Yes	No		Yes	3
Kodama *et al.*, 2009^16^	F	39	No	Yes	Yes	Yes	No	No	Yes	Yes	No		Yes	15
Kodama *et al.*, 2009^16^	F	57	Yes	Yes	Yes	No	Yes		Yes	Yes	No		Yes	15
McGee *et al.*, 2020^17^	F	81	No	Yes	Yes	No	Yes	Yes	Yes	Yes	No		Yes	9
Morawiec *et al.*, 2017^18^	F	82	Yes	Yes	Yes	Yes	No	Yes	Yes	Yes	No		Yes	10
Nadeem *et al.*, 2021^19^	F	89	Yes	Yes	Yes	No	Yes	Yes	Yes	Yes	No		Yes	1
Rizly *et al.*, 2024^20^	F	84	No	Yes	Yes	No	Yes	Yes	Yes	Yes	No		Yes	1
Limm *et al.*, 2014^4^	F	80	Yes	No	No			Yes	Yes	Yes	No		Yes	1
Current case report	F	64	No	Yes	Yes	No	Yes	Yes	Yes	Yes	Yes	2	No	6

**Table 2:** Characteristics of patients having atrioventricular block with Takotsubo cardiomyopathy in literature

	AV conduction restoration	AV block persistence
Number of patients	7 (31.8%)	15 (68.2%)
Age, y	61 [61–64]	80 [61–83]
Female	7/7 (100%)	15/15 (100%)
Chest pain at initial presentation	3/7 (42.9%)	7/15 (46.7%)
Syncope at initial presentation	5/7 (71.4%)	11/15 (73.3%)
QRS enlargement	5/7 (71.4%)	9/14 (64.3%)
If QRS enlargement, LBBB	1/5 (20%)	3/9 (33.3%)
If QRS enlargement, RBBB	4/5 (80%)	6/9 (66.7%)
Troponin I or T elevation	5/7 (71.4%)	11/15 (73.3%)
Initial LVEF alteration	7/7 (100%)	15/15 (100%)
LVEF restoration	7/7 (100%)	14/15 (93.3%)
Time to sinus restoration, d	30 [2–51]	—
Early sinus restoration (<7 days)	3/7 (42.9%)	—
Pacemaker implantation	4/7 (57.1%)	15/15 (100%)
Time to pacemaker implantation, d	6 [1–7]	5 [2–13]

Data are gathered from the cases review presented in *Table 1*. Data are presented as number (percentage) or median [25–75% interquartile].

AV, atrioventricular; LBBB, left bundle branch block; LVEF, left ventricular ejection fraction; RBBB, right bundle branch block.

Given the predominance of irreversible AV block in our review, pacemaker implantation is often considered a pragmatic strategy. However, our review suggests that early systematic pacemaker implantation is to be nuanced, particularly in younger patients which may restore sinus rhythm early. Prolonged temporary pacing as an alternative strategy was considered in this case but ultimately deemed inappropriate because the patient was entirely pacemaker-dependent prior to permanent implantation and was a foreign national visiting France. Five of the 7 patients who recovered from AV block experienced delayed recovery between one and three months, indicating that a late sinus rhythm restoration is still possible. The absence of predictive factors of sinus rhythm restoration emphasizes the difficulty in anticipating evolution; pacemaker implantation appears justified in most cases. In the present case, persistent LBBB may be explained by two mechanisms: (1) incomplete resolution of TCM-related myocardial oedema involving the basal septum and the left bundle branch region, such spatial dissociation between myocardial and conduction system recovery has been described and may account for delayed or incomplete normalization of intraventricular conduction; (2) persistent LBBB may be related to a preexisting conduction disease, precipitated or revealed by TCM. Unfortunately, the absence of prior ECGs and limited follow-up beyond one month do not allow discrimination between these two hypotheses. Persistent LBBB after TCM recovery suggests residual conduction system injury and supports the hypothesis of incomplete electrical recovery despite normalization of LVEF. Moreover, LBBB is associated with a risk of progression to advanced AV conduction disease and ventricular desynchrony, particularly in elderly patients. Its persistence reinforces the clinical relevance of permanent pacemaker implantation. To date, there are no specific recommendations on the management of conduction disturbances associated with TCM and further research is needed to optimize the therapeutic strategies.

## Conclusion

In TCM patients presenting with complete AV block, a tailored approach is essential. While most cases will ultimately require pacemaker implantation due to persistent conduction disturbance, 10% of patients may experience spontaneous early sinus rhythm restoration. To date, there are no specific recommendations on the management of conduction disturbances associated with TCM, and further research is needed to optimize the therapeutic strategies.

## Data Availability

The data underlying this case report can be made available upon reasonable request to the corresponding author.

## Lead author biography

Sacha Jourdin is a cardiology resident.

## Notes

**Consent:** The authors confirm that written consent for submission and publication of this case report, including images and associated text, has been obtained from the patient in line with COPE guidance.
